# Neurally adjusted ventilatory assist in pediatrics: why, when, and how?

**DOI:** 10.5935/0103-507X.20170064

**Published:** 2017

**Authors:** Lívia Barboza Andrade, Rodrigo Guellner Ghedini, Alexandre Simões Dias, Jefferson Pedro Piva

**Affiliations:** 1 Instituto de Medicina Integral Prof. Fernando Figueira) - Recife (PE), Brazil.; 2 Pediatric Intensive Care Unit, Hospital de Clínicas de Porto Alegre), Universidade Federal do Rio Grande do Sul - Porto Alegre (RS), Brazil.

## Introduction

In pediatrics, good synchrony in controlled assisted ventilation is not always possible and may delay recovery, prolong mechanical ventilation (MV), and contribute to loss of muscle strength and increased calorie expenditure.^(1)^

In controlled assisted ventilation, the trigger (drive) is a decisive factor in the release of the assisted cycle, as it is regulated by the pressure difference or flow difference in the system. Very sensitive triggers induce hyperventilation and atrophy of respiratory muscles, whereas less sensitive systems require more effort, inducing hypoventilation, excessive energy expenditure, and discomfort. Even with adequate sensitivity, there is a delay in the release of the assisted cycle resulting from the interval between the central nerve impulse and the respiratory muscle contraction to initiate the trigger. Air leakage around the tracheal tube is a limiting factor that may not be perceived or compensated for by the device, requiring even greater effort by the child.

Neurally adjusted ventilatory assist (NAVA; Maquet^®^, Sweden) is a minimally invasive technology that releases proportional pressure cycling in response to electrical activity of the diaphragm (EAdi), adapting ventilatory support to the patient's actual demand.^(2)^ Thus, the patient, through his neural drive, regulates the frequency of cycles and the volume to be released in each of them, with the benefits of avoiding hyper- or hypoventilation of support, preserving the EAdi, increasing the interaction with the ventilator, not being influenced by air leaks around the tracheal tube, and, especially, incorporating the natural variability of breathing. In short, the mechanical ventilator in the NAVA mode divides the load with the diaphragm to support the ventilation in a synchronized and proportional way and can be used in an invasive or non-invasive way (NIV-NAVA).

The NAVA mode was first used in Latin America in 2009 with an adult population, and since then, only 15 pediatric studies have been published, where the neonatal population predominates.^(2,3)^ Justifications for underutilization of the NAVA mode in pediatric intensive care units (ICU) include theoretical and unconfirmed concepts in large studies, high cost, lack of reference values for levels of electrical activity, and the impact of this monitoring and the ventilatory strategy on clinical outcomes. There is a lack of understanding of the ventilatory and monitoring possibilities that this tool can offer the clinician at the bedside.

## Why monitor the electrical activity of the diaphragm?

In spontaneous ventilation, the tidal volume generated is proportional to the intensity of contractility of the respiratory muscles, especially the diaphragm. The intensity of this contraction results from the interaction of several factors: afferent information on lung inflation and deflation, arterial gases, and diaphragm contractile capacity (sedation and atrophy), among others. Therefore, the neural respiratory drive identifies and responds to various factors, generating an EAdi proportional to the ventilation requirements. The EAdi signal is measured and used to trigger the assisted inspiration, releasing an inspiratory pressure proportional to the electrical activity. The ventilatory cycle ends when a 30% reduction in the EAdi peak is observed.^(2)^ This allows for synchronization between the electrical activity of the patient and the pressure generated in the ventilator in terms of time and proportionality.

**Figure 1:**
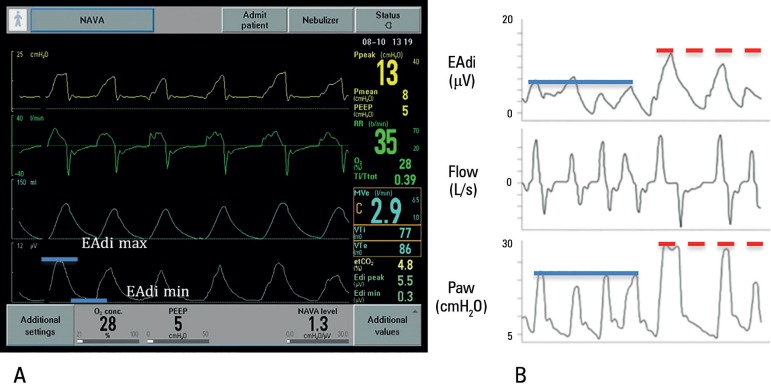
Demonstration of pressure, flow, and electrical activity curves of the diaphragm. (A) Maximum electrical activity of the diaphragm, showing phasic activity of the diaphragm, and minimal electrical activity of the diaphragm, or tonic activity. (B) Proportional increase in airway pressure in response to the corresponding increase in electrical activity of the diaphragm. EAdi - electrical activity of the diaphragm; flow - flow; paw - airway pressure.

Source: Adapted from Stein H, Firestone K, Rimesberg P. Synchronized mechanical ventilation using electrical activity of the diaphragm in neonates. Clin Perinatol. 2012;39(3):525-42.^(5)^

Regardless of ventilation under NAVA, the EAdi waveform can be used to monitor neural respiratory rate, which presents a cyclic characteristic with a pattern of variations between maximum (phasic EAdi) and minimum values (tonic EAdi), whose mean in infants and children varies between 8 and 20 microvolts, with a tendency to higher values in non-invasive modes^(2,4)^ (Figure 1).

Tonic EAdi persists until the end of expiration above the baseline and is usually absent in healthy adults and children older than 1 year old. In newborns and infants, it is higher to actively maintain the lung volume at the end of the expiration above the volume of relaxation, thus preventing alveolar derecruitment. Other mechanisms are involved in this process, such as rapid respiratory rate with short expiratory time and delayed expiratory flow by constriction of the larynx. In intubated children, the tracheal cannula prevents laryngeal braking, further reinforcing the importance of the tonic activity of the diaphragm, which can be evaluated continuously through the minimal EAdi.^(6)^

Neural inspiratory efforts (sighs), as well as periods of flat EAdi during central apnea, can also be observed. Compared with adults, the signal in children shows high variability, with a higher tonic activity in pre-term and in non-invasively ventilated patients.

A large study in Canada evaluated EAdi in children undergoing conventional ventilation, in the acute phase of the disease, in the pre- and post-extubation period, and in the ICU discharge. Peak EAdi values were markedly suppressed in the acute phase (3.6µV) and increased to 4.8µV in the pre-extubation period. There were periods of total diaphragm inactivity in the acute phase, even with low levels of care. Shortly after extubation, the EAdi increased to 15µV and remained high (13 - 15µV) until discharge from the ICU. Children with lung disease had higher electrical activity, while low EAdi in the acute phase may be caused by the use of sedation and over-assistance of MV.^(7)^

In Finland, EAdi was measured in 81 children (with lung disease and post-surgery) in NAVA mode and 1 hour post-extubation. When ventilated, the NAVA level was adjusted to maintain peak EAdi between 5 and 15µV. Children with pulmonary disease presented higher EAdi levels than post-surgical patients at all stages of treatment. After extubation, children with pulmonary disease have, on average, 20µV compared to post-surgery children, who presented 9µV.^(8)^

There are several citations in cross-sectional studies and case series on EAdi monitoring, such as in cases of diaphragm paralysis, central hypoventilation, preterm weaning, infants with viral bronchiolitis, children with difficult weaning, and respiratory control disorders.^(2,9)^ Monitoring the EAdi allows clinicians to adapt ventilatory parameters in an individualized way, avoiding the over-assistance and consequent diaphragm atrophy (injury due to disuse). The increase in peak EAdi levels suggests insufficient ventilatory support; in contrast, a strong tonic activity may reflect the child's effort to increase his lung volume.

## NAVA mode: mean airway pressure and lung protection

In spontaneous breathing, as lung inflation progresses, pulmonary stretch receptors behave as sensors that inform adequate inspiratory volume and "turn off" inspiration.

In NAVA mode, in which neural inspiration also controls delivery of care, the ventilatory cycle may be discontinued when neural exhalation begins. Some studies show that in spontaneous breathing, children have lower airway mean pressures and tidal volumes very similar to those found in NAVA mode.^(10-12)^ The justification for this behavior is the reflex control of the ventilator, which promotes better comfort and synchronization due to lower electrical activity and, consequently, lower mean pressure.

**Figure 2:**
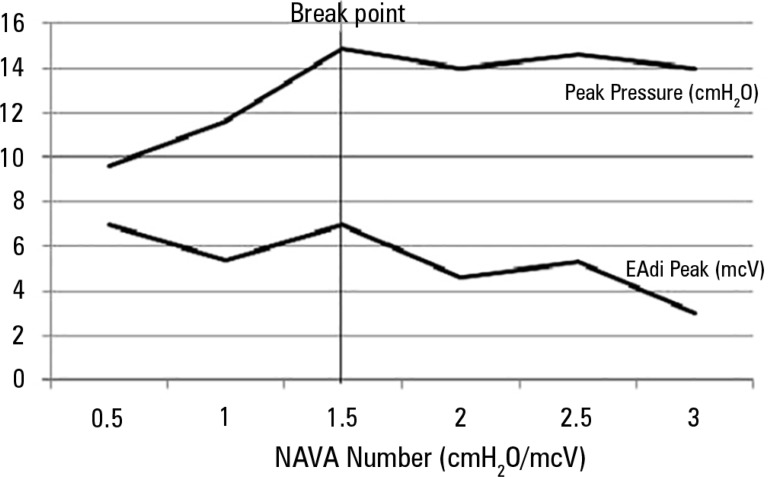
Increase in the positive inspiratory pressure and the electrical activity of the diaphragm according to the level of neurally adjusted ventilatory assist increases until the breaking point (1.5cmH_2_O/mcV) in premature infants. NAVA - neurally adjusted ventilatory assist.

Source: Stein H, Alosh H, Ethington P, White DB. Prospective crossover comparison between NAVA and pressure control ventilation in premature neonates less than 1500 grams. J Perinatol 2013;33(6):452-6.^(13)^

In one study, premature infants presented downregulation of EAdi to avoid overdistension when submitted to a gradual increase in the NAVA level (0.5cmH_2_O every 3 minutes) until reaching 4cmH_2_O. In the initial portion of the experiment, an increase in the positive inspiratory pressure (PIP) proportional to the increase in assistance was observed, which occurred to a certain extent where the pressure did not increase. The authors (Figure 2) called this point a breakpoint (a plateau was observed in the PIP). The behavior of tidal volume also followed a similar pattern.^(13)^

Another study showed reduced respiratory muscle load and lower PIP when premature infants were ventilated in NAVA mode compared to synchronized intermittent mandatory ventilation (SIMV) associated with pressure support ventilation.^(14)^

The reduction of the pressures observed in the abovementioned studies was associated with the reduction of the partial pressure of carbon dioxide (PaCO_2_), the improvement of oxygen partial pressure/inspired oxygen fraction (PaO_2_/FiO_2_), and the time of weaning, without hemodynamic impact.

## Variability of breathing

In contrast to constant ventilation in conventional modes, the variability of pressures and volumes in neural ventilation is high, as it reflects the respiratory center output.^(15)^ Biological systems are characterized by their intrinsic variability, called noisiness, which is opposed to monotonic behaviors observed in mechanical systems. Reduction in respiratory variability is associated with adverse outcomes.^(15,16)^

One study compared NAVA, pressure-controlled ventilation, and pressure support ventilation (PSV) in children, and EAdi was measured continuously and its variability assessed by an index that registered the rhythmicity of the respiratory pattern compared to healthy controls in spontaneous breathing. NAVA was the mode that presented greater variability, resembling the controls.^(15)^ In children who were sick, greater comfort was also observed when ventilated in NAVA, instead of PSV; still, there was better synchrony, reduction of ventilatory drive, and increased respiration variability.^(17)^

## Patient-ventilator interaction

Asynchrony between the patient and ventilator is considered an important cause of cyanotic episodes and can result in large tidal volumes, air trapping, blood pressure fluctuations, and worsening of oxygenation. Similar to what occurs in adults, 16 studies involving infants and children observed that the interaction is better in NAVA mode compared to controlled modes. However, asynchrony indices are quite varied in these studies: 12 to 73% in conventional modes compared to 0 to 20% in NAVA mode.^(2)^ This better assistance is due to more sensitive and accurate drive mechanisms, correct cycling, and proportionality of effort assistance.

## Practical aspects in the use of NAVA in pediatrics

**Figure 3:**
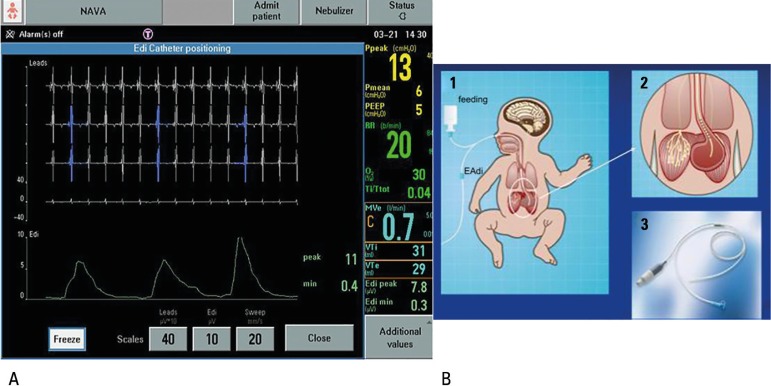
(A) Lines marked in blue on the electrocardiographic tracing demonstrate adequate positioning of the catheter for measuring diaphragm electrical activity. (B) Simultaneous recording of electrical activity. (1) Schematic of the positioning of the catheter and its outputs for feeding and coupling with the neurally adjusted ventilation assist cable. (2) Probe in the esophagogastric position. (C) Neurally adjusted ventilation assist cable that attaches to the mechanical ventilator.

Source: Adapted from Stein H, Firestone K, Rimesberg P. Synchronized mechanical ventilation using electrical activity of the diaphragm in neonates. Clin Perinatol 2012;39(3):525-42.^(5)^

The EAdi signal is picked up by electrodes embedded in the distal part of the catheter, positioned at the level of the crural diaphragm. The passage of the catheter has been described as safe and easy, allowing its use for infusion of diet, without interfering in the signal quality.^(18,19)^ In the insertion, it is suggested to use the measurements of the distances between the nose, the lobe of the ear, and xiphoid appendix in the formula indicated by the manufacturer. The catheter is adequate when the central electrode is at the height of the diaphragm and is visible on the ventilator screen with the presence of blue signals in the central curves (Figure 3).

After confirmation of the positioning, with good capture of the EAdi signal, titration of the NAVA level begins, and minimum assistance values between 0.5 and 2cmH_2_O/µV (up to 4 in children) are recommended. Lower values are not interpreted as ventilatory drives. The magnitude of the mechanical assistance varies with each breath according to the EAdi and the gain factor (NAVA level). In practical terms, the "NAVA level" is the factor to be multiplied in the EAdi to generate a certain inspiratory pressure. Setting a very low NAVA level requires an excessive diaphragm load to generate PIP, while high NAVA values require less effort and induce muscle atrophy. The mathematical equation of the relationship between PIP and EAdi can be expressed as follows:

**Table:** 

PIP = [Level of NAVA x ∆ EAdi (max - min)] + PEEP

The target EAdi peak should be between 5 and 15µV, considering the breathing fluctuations. Thus, positive end-expiratory pressure (PEEP), FiO_2_, and NAVA level are the only predefined parameters. For safety, upper pressure limits must be defined and backup ventilation must be ready, which automatically activates if EAdi does not occur.

## Pediatric studies

Table 1 summarizes the main pediatric studies comparing NAVA with pneumatic ventilatory modes. No studies with NIV-NAVA were included.

**Table 1:** Pediatric studies investigating the use of neurally adjusted ventilatory assist in an invasive manner compared with controlled ventilation in pneumatic modes

Author	Number of patients	Type of study	Outcomes	Results
Clement et al.^(9)^	33	Crossover	Ventilator response time, inspiratory efforts, and breathing work	NAVA demonstrated a shorter response time, reduced trigger, reduced workload (lower pressure/time product)
Alander et al.^(11)^	18	Crossover	Index of asynchrony (analysis of ineffective efforts and self-trigger), analysis of airway pressures, vital signs	IA (NAVA) = 08 IA (CMV) = 28 Lower PIP and MAP
de la Oliva et al.^(17)^	12	Non-randomized crossover	Index of asynchrony (ineffective effort and self-trigger analysis), respiratory variability, COMFORT score	IA (NAVA) = 2 IA (CMV) = 12 Better variability and comfort scores
Breatnach et al.^(20)^	16	Crossover	Asynchrony (trigger and cycling), analysis of airway pressures	Better synchrony, reduced PIP and MAP levels in NAVA mode
Bordessoule et al.^(21)^	10	Case series	Index of asynchrony (ineffective effort and self-trigger analysis), respiratory variability	IA (NAVA) = 11 IA (CMV) = 25 NAVA has greater variability of EAdi that is brought about in ventilator pressure variability
Vignaux et al.^(22)^	19	Crossover, randomized, prospective	Index of asynchrony (analysis of ineffective efforts and self-trigger)	IA (NAVA) = 4 IA (CMV) = 29
Kallio et al.^(23)^	170	Randomized clinical trial	Ventilation time, ICU stay, required amount of sedation, ventilation parameters	Lower MV time and pediatric ICU stay. Sedation was lower in NAVA in clinical patients (no significance in surgical patients). Lower FiO_2_ and PIP

NAVA - neurally adjusted ventilatory assist; IA - index of asynchrony; CMV - conventional mechanical ventilation; PIP - positive inspiratory pressure; MAP - mean airway pressure; EAdi - electrical activity of the diaphragm; ICU - intensive care unit; FiO_2_ - inspired oxygen fraction.

## Final comments

Current studies indicate that neural ventilation in infants and children is better tolerated compared to conventional ventilatory modes. It appears to be safe, it has better patient-ventilator interaction, provides comfort, requires a lower level of sedation, shortens length of stay, and offers monitoring of electrical activity. However, its long-term role is still uncertain, especially regarding the duration of mechanical ventilation, length of stay, and mortality in children.
